# Volatile Organic Silicon Compounds in Biogases: Development of Sampling and Analytical Methods for Total Silicon Quantification by ICP-OES

**DOI:** 10.1155/2014/537080

**Published:** 2014-10-15

**Authors:** Claire Chottier, Vincent Chatain, Jennifer Julien, Nathalie Dumont, David Lebouil, Patrick Germain

**Affiliations:** Laboratoire de Génie Civil et d'Ingénierie Environnementale LGCIE, Université de Lyon, INSA-Lyon, 20 Avenue Albert Einstein, 69621 Villeurbanne, France

## Abstract

Current waste management policies favor biogases (digester gases (DGs) and landfill gases (LFGs)) valorization as it becomes a way for energy politics. However, volatile organic silicon compounds (VOSiCs) contained into DGs/LFGs severely damage combustion engines and endanger the conversion into electricity by power plants, resulting in a high purification level requirement. Assessing treatment efficiency is still difficult. No consensus has been reached to provide a standardized sampling and quantification of VOSiCs into gases because of their diversity, their physicochemical properties, and the omnipresence of silicon in analytical chains. Usually, samplings are done by adsorption or absorption and quantification made by gas chromatography-mass spectrometry (GC-MS) or inductively coupled plasma-optical emission spectrometry (ICP-OES). In this objective, this paper presents and discusses the optimization of a patented method consisting in VOSiCs sampling by absorption of 100% ethanol and quantification of total Si by ICP-OES.

## 1. Introduction

**Figure 1:**
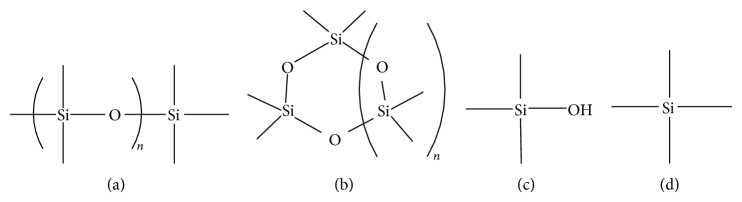
Structural formulas of VOSiCs: (a) linear (1 < *n* < 3), (b) cyclic (1 < *n* < 5), (c) trimethylsilanol (TMSol), and (d) tetramethylsilane (TMS).

Biogases (digester gas (DG) and landfill gas (LFG)), issued from anaerobic digestion of wastewater, sewage sludges, or wastes, could be an answer to the lack of energy, and at the same time, could decrease fossil energy consumption and avoid greenhouse gas emissions. Benefits of this renewable energy lead scientists to optimize biogas valorization. However, our daily life and industrial wastes contain silicone polymers or low molecular weight silicones [1] that end in wastewater treatment plants (WWTPs) [2], in landfills or in waste methanization facilities. Also, during the anaerobic waste degradation, silicones and other silicon-containing materials (detergents, soaps, etc.) generate volatile organic silicon compounds (VOSiCs, including siloxanes). Silicon present in biogas originates mainly from those compounds, which are known to be volatile compared to Si mineral. Among them different types of structures could be discriminated, and the most studied are the methyl siloxanes. However, silanols (compounds containing the Si–OH group), such as trimethylsilanol (TMSol), silanes (Si_*n*_H_2*n*+2_), such as tetramethylsilane (TMS), or other organic molecules can also be found in biogases [3]. Their structural formulas are shown in Figure 1.

The main cyclic VOSiCs present in biogases are the octamethylcyclotetrasiloxane (D4), the decamethylcyclopentasiloxane (D5), the hexamethylcyclotrisiloxane (D3), and to a lesser extent the dodecamethylcyclohexasiloxane (D6). The main linear VOSiCs are the trimethylsilanol (TMSol), the hexamethyldisiloxane (L2), the octamethyltrisiloxane (L3), and the barely present decamethyltetrasiloxane (L4) [4, 5]. Depending upon the type, origin, and quality of organic waste landfilling, sewage sludge digestion, or sorted biowaste digestion processes, relative proportions of VOSiCs can fluctuate [6].

During combustion, VOSiCs are oxidized into silica and silicates, which deposit in combustion chambers [3, 4, 7, 8]. The accumulation of those abrasive deposits to a thickness of several millimeters affects equipment's performances (motor, spark plugs, pistons, cylinder heads, valves, etc.) and contaminates lubricating oils, resulting in an increasing global cost of maintenance and cleaning [9]. Various abatement techniques, such as solvent wash and adsorption on solids, have been developed or adapted to remove those harmful trace constituents from biogas [9–13]. To design and subsequently assess the efficiency of those techniques, a reliable analysis of VOSiCs for DGs/LFGs is required. Previously, there has been no standardized protocol for VOSiCs quantification. First studies have revealed that results can significantly vary depending on the sampling and screening techniques [5, 14, 15]. One of the foremost methods is based on gas chromatography coupled with mass spectrometry (GC-MS), which allows the speciation of VOSiCs [3, 8, 10]. Among hundreds of existing VOSiCs, only 6 to 10 compounds are usually quantified by GC-MS for various reasons; some are better known, more common and/or standards are available. Due to the unavailability of certain analytical standards, most laboratories provide results as toluene (or other) equivalents. Peak areas on chromatograms are reported to a toluene calibration curve in order to derive a numerical value of concentration. Several disadvantages dependent on the analytical chain, linked to the storage, the transport or even the availability of analytical standards, disturb this speciation technique which is nowadays the most employed.

Another technique uses inductively coupled plasma-optical emission spectrometry (ICP-OES) to allow a global quantification of the total silicon content in biogases [7, 16]. Thanks to the use of an absorption method based on an easily transportable device [16], VOSiCs can be quickly and directly trapped (in less than 25 min) into absorbing solutions. All VOSiCs are soluble in various organic solvents, such as oil [9], toluene, acetone, heptane, hexane [17, 18], and methanol [19, 20]. However, some major analytical problems have been highlighted when elementary Si from VOSiCs is analyzed by ICP-OES. For example, the Si content of TMSol aqueous solutions is overestimated by a 17 factor in comparison to the classical Si mineral NIST standard [21]. Hagmann et al. [17] also have shown that Si contents of L2, D3, and D4 solutions in organic solvent are overestimated, respectively, by 8.7, 3.6, and 1.4 in comparison to octaphenylcyclotetrasiloxane standard. Sánchez et al. [22] have shown that it goes the same way for the Si contents of VOSiCs in xylene matrices in comparison to dimethyloctylchlorosilane standard. For example, D4 Si signal is exacerbated by a factor 1.5 and L2 Si signal by a factor 17. So, this phenomenon could occur with any matrices and with the others VOSiC present in biogases. Hagmann et al. [17] and Sánchez et al. [22] mentioned that the origin of the overestimation takes place during the nebulization step in the ICP-OES apparatus. Volatile compounds could desorb outside of the mist and enhance silicon level in the outside atmosphere. In this case, it is a source of analytical bias, which systematically overestimates Si amounts.

Some scientists use both methods to exploit their complementarity. Schweigkofler and Niessner [4] proposed a GC-MS/AES as a detection coupling; VOSiC identification is allowed by the mass spectrometer whereas quantification is performed by atomic emission spectrometry. Chao [23] used GC, to separate VOSiCs, coupled to an atomic emission detector using a microwave-induced He plasma to perform quantification.

This research paper presents and discusses results obtained on several biogases, in using ethanol to absorb VOSiCs and then ICP-OES to quantify total elementary Si. Laboratory development thanks to synthetic matrices and fieldwork validations thanks to biogases sampled on sites will be established to evaluate the efficiency of the analytical methodology developed to overcome the issue of Si overestimation during ICP-OES analyses of VOSiC in organic matrices.

## 2. Materials and Methods

### 2.1. Reagents and Solutions

Hexamethyldisiloxane (98.5%), octamethyltrisiloxane (97%), decamethyltetrasiloxane (97%), dodecamethylpentasiloxane (97%), hexamethylcyclotrisiloxane (98%), octamethylcyclotetrasiloxane (98%), and decamethylcyclopentasiloxane (97%) were purchased at Sigma-Aldrich; dodecamethylcyclohexasiloxane (97%) was purchased at ABCR, trimethylsilanol (99.3%) was purchased at Chemos, and absolute ethanol (99.9%) was purchased at Prolabo. All stock solutions and dilutions were made into absolute ethanol. All VOSiCs standards were tested in comparison to a L5 calibration curve, either alone at 0.5, 1, 4, and 5 mgSi/L to assess their individual analytical response in ethanol matrices, or via 300 mgSi/L mixtures showing different VOSiCs distributions diluted to reach 2, 4, and 5 mgSi/L. Individual VOSiC analytical responses are named afterwards as “response factors.” Response factor is defined as the measured Si concentration (in comparison to L5 calibration curve) over the calculated Si concentration by dilution of the standard.

### 2.2. Real Samples Origins

LFGs produced at 3 French nonhazardous waste landfills (landfill A, B, and C) and DGs produced at 2 French WWTPs (WWTP A and B) were sampled between the conditioning system and a potential pretreatment.

### 2.3. Sampling and Analysis

#### 2.3.1. Bags Sampling

Tedlar bags of 3 liters with polypropylene fittings were used to sample and store LFGs and DGs. Samples were sent to a private accredited laboratory, able to perform a speciation of VOSiCs by GC-MS analysis. The analysis procedure consists of the direct injection of gaseous samples from bags into the GC-MS device. Laboratory provides VOSiCs concentrations in mg of each analyzed compound per Nm^3^ of dry biogas. The analytical relative uncertainty provided by the laboratory is of 15% for each compound.

#### 2.3.2. Liquid Absorption Sampling

**Figure 2:**
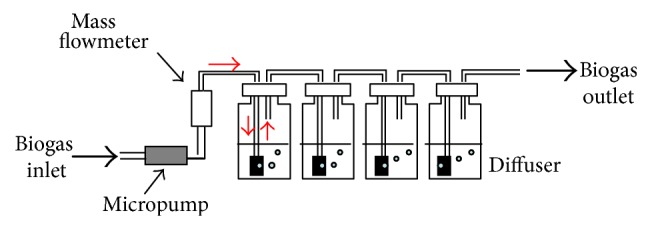
Principle of the liquid absorption bubbling device.

The patented method used and developed by Germain et al. [16] is based on a known biogas volume, bubbling at a controlled flow rate thanks to a mass flowmeter (Brooks) calibrated for biogas, into liquid solutions able to absorb VOSiCs. In order to avoid any contamination, a Si-free sampling device has been built (Figure 2).

The absorption device consists of four successive 250 mL HDPE bottles (Azlon) each filled with 150 mL of absolute ethanol. After sampling, bottles are stored at 4°C until analysis.

Samples analyses from each bottle are performed by a radially observed Ultima 2 Horiba Jobin-Yvon ICP-OES (Longjumeau, France) running through an argon flow (4.5; Linde Gas). The apparatus is functioning with a 40.68 MHz radiofrequency generator and a Czerny-Turner grating monochromator. The classical cyclonic spray chamber has been replaced by an IsoMist (Glass Expansion, Australia), which is a programmable temperature cyclonic spray chamber (variable from −10°C to +60°C). The IsoMist allows pure ethanol injection without plasma extinction by decreasing the temperature in the nebulization chamber to −10°C. Moreover this will have a side benefit which is the decrease of analyte volatilization outside of the mist drops and a reduction of the induced overestimation. Si concentrations are determined at 251.6 nm with a viewing height above load coil of 5 mm and a radial plasma viewing mode. The torch was vertical, demountable with a 3 mm i.d. injector. The radiofrequency power used was of 1400 W, the sample uptake was of 0.5 mL/min, the nebulisation pressure was of 1 bar, and plasma gas flow rate and sheath gas flow rate were, respectively, of 16 and 0.35 L/min.

Calibration, ranging from 0 to 5 mgSi/L, is done with L5 standards, a nonvolatile siloxane (vapor pressure < 0.01 kPa at 25°C), logically absent from DGs and LFGs.

Considering the Si levels in the absorbing solutions, the volume of solvent used and the volume of biogas in contact, it is possible to derive the Si content into mgSi/Nm^3^ of biogas.

## 3. Results and Discussion

### 3.1. LFGs and WWTPs DGs VOSiCs Composition

Table 1 provides a summary of VOSiCs speciation from LFGs and WWTPs DGs sampled in different fieldworks and at different times during the year.

It is noticeable that VOSiCs composition varies in time and space, which will direct our methodology development. Main VOSiCs are different in LFGs (D4, TMSol, D5, and L2) than in WWTPs DGs (D5, D4); as well some VOSiC can be totally absent from one site and in large quantity in other sites. We can cite as examples the cases of TMSol or L2, which are residual in WWTPs DGs and significant in LFGs. About L5 quantification, analyses reveal that its level stays under the detection limit of the device (0.005 mg/Nm^3^) whatever the sampling site.

### 3.2. Development of the ICP-OES Analytical Methodology for VOSiC Total Si Measurements

#### 3.2.1. VOSiCs Individual Analytical Standard Deviation

**Figure 3:**
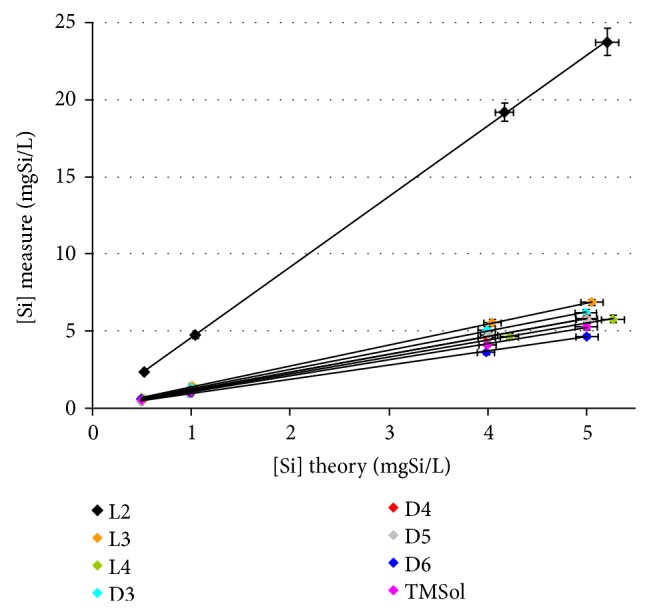
VOSiCs ICP-OES analytical responses (L5 calibration curve) by comparison with the theoretical concentration. Analyses are performed at −10°C and each value is the mean of 3 measurements.

Figure 3 shows Si measurements for each VOSiCs standard solutions (using L5 calibration curve) on the calibration range routinely used in the analysis procedure, namely, from 0.5 to 5 mgSi/L as a function of the theoretical calculated concentration of each standard solution. In this case, the response factor is equal to the slope for each VOSiC regression line on the studied range. Uncertainties on the *x*-axis correspond to standard solution preparation and on the *y*-axis to the standard deviation over 3 measurements of the solution.

Linearity of VOSiCs analytical individual response in ethanol is highlighted (*r* ^2^ ranging from 0.9993 to 1). Therefore, it verifies VOSiCs solubility in ethanol in the range from 0.5 to 5 mgSi/L. Furthermore, 7 out of 8 VOSiCs show an individual response factor (equal to the slope for each VOSiC regression on the studied range) close to 1 (considered VOSiC ICP-OES responds as the L5 standard), namely, between 0.9 and 1.4. Only L2 presents a remaining high response factor of 4.6. It is linked to the structure of L2 and its high vapor pressure (*ca.* 5.5 kPa at 25°C), which consequently facilitates its volatilization and increases analytical bias.

According only to vapor pressure, TMSol must show an important residual overestimation in these conditions as its vapor pressure (*ca.* 9.9 kPa at 25°C) is higher than the one of L2. However, the response factor of TMSol is only around 1.1 which is linked to Henry's law through the Henry's law constant which is function of temperature, solute and solvent nature. Indeed, this is explained by the hydrogen bonds between the hydroxyl groups of TMSol and those of ethanol. This phenomenon improves the solute/solvent interactions and annihilates TMSol desorption from the mist drops at low temperature.

Postanalysis adjustments need to be performed on ICP-OES raw data interpretation as no other technical improvement is available to further decrease the temperature in the nebulization chamber and avoid L2 desorption from mist.

#### 3.2.2. Method Adjustment via Synthetic Laboratory Solutions

As explained above, L2, particularly substantial in LFGs, disturbs the accuracy of the ICP-OES analytical method in ethanol matrix. L2 percentage can fluctuate from one site to another, ranging, as shown in Table 1, from 10 to 19% of the total Si LFGs content and be totally absent in WWTPs DGs.

To remain consistent, mixtures of commercial VOSiCs standards (TMSol, L2, L3, D3, D4, and D5) in ethanol, modeled after typical LFGs and DGs GC-MS analyses, have been simulated and analyzed in laboratory by ICP-OES. Five stock solutions of synthetic mixtures have been made with a total Si concentration of 300 mgSi/L. The difference between these 5 mixtures is the Si percentage coming from L2 which was set up at 10, 15, 20, and 25%, proportions of the other VOSiCs are also evolving but ratios remain constant compared to each other. For each synthetic mixture, 3 standards (at 2, 4, and 5 mg of total Si per liter) from stock standard solutions dilution, covering the whole range of L5 calibration, have been realized. The mean ICP-OES response factors between the 3 total Si concentrations for each percentage of L2 are reported in Table 2.

Different percentages of L2, covering classical contents reached in LFGs, have been applied on a typical LFG composition copy, causing a modification of the other VOSiCs silicon amounts represented. The evolution of response factors is linear as a function of the Si percentage coming from L2. A straight line (*y* = 0.0284*x* + 1.0027) with a correlation coefficient *r* ^2^ of 0.9932 is obtained. Moreover, it is noticeable that, for a same Si percentage coming from L2, whatever the mixture composition in terms of total Si amount (2, 4 or 5 mgSi/L), response factors are constant (0% < RSD < 7%).

When L2 is absent from the analyzed mixtures, the ICP-OES result in comparison to L5 calibration curve is accurate (at 0% of L2: response factor of 1.0), which confirms the accuracy of the sampling and analytical methods for WWTPs DGs, without any adjustment. As the Si level coming from L2 is higher than 10% for the other solutions, the application of a correction by the mean response factor will only concern LFGs. A global theoretical response factor of 1.5 ± 0.2, covering the whole deviations observed for L2 proportions representative of real LFGs, is established from these results.

### 3.3. Method Validation for LFG Analysis

Only one validation is presented, the methodology is applicable to all LFG analyses (with a classical L2 percentage less than 25% of the total Si LFGs content).

#### 3.3.1. Experimental GC-MS and ICP-OES Results on LFG B3

Successively, via the absorption device, set up with 4 bottles containing ethanol, and a via 3L Tedlar bag, biogas has been directly sampled from a single tapping point installed upstream from the combustion engine at landfill B to provide a gaseous sample for GC-MS analysis (Table 3) and a liquid sample for ICP-OES analysis (Table 4) which will be compared.

For LFG B3, the GC-MS quantification provides a concentration of 29 ± 5 mgSi/Nm^3^. This result will be named* Result 1* afterwards. The ICP-OES quantification with the application of the 1.5 ± 0.2 correction factor (deduced by laboratory experiments) leads to a concentration ranging from 28 to 36 mgSi/Nm^3^ for LFG. This result will be named* Result 2* afterwards.

The global relative uncertainty of experimental determination of the total Si amount by ICP-OES in LFG has been calculated and associated with a potential margin of error of 15% and the relative uncertainty calculated for total Si amount provided by GC-MS is also of 15%.

#### 3.3.2. Theoretical ICP-OES Result Calculation in mgSi/Nm^3^ LFG

A theoretical calculation of the Si total amount has been carried out. It took into account the percentage of Si coming from L2 furnished by the GC-MS analysis (see Table 1, LFG B3, %Si (L2) = 19%; which is the same analysis to the one presented in Table 4) and the L2 individual response factor in ethanol matrix, that is, 4.6 (see Figure 3). Equation (1) summarizes the different adjustments (due to the operational conditions and L2 theoretical adjustments) to apply to convert the Si level, furnished in mgSi/L of ethanol by the ICP-OES device, in mgSi/Nm^3^ of biogas:

(1) $$\begin{array}{c} {\left[\text{Si}\right]}_{\text{mg}/\text{N}{\text{m}}^{3}}=\frac{a}{1+\left(3.6\times \left(b/100\right)\right)}\times \frac{c}{d}\times 1000. \end{array}$$

The terms of (1) are described as follows: *a* (mgSi/L): sum of the raw Si concentration in the 4 bottles of the sampling device measured by ICP-OES with L5 calibration. *b* (%): percentage of Si coming from L2 within the total Si determination by GC/MS analysis. *c* (L): volume of ethanol in one bottle of the sampling device. *d* (L): volume of LFG sampled.

The relative uncertainty on the result of the calculated theoretical ICP-OES value has been calculated and is of 15%.

In our example, the application of (1) leads to a theoretical ICP-OES value for LFG B3 of 28 ± 5 mgSi/Nm^3^. This result will be named* Result 3* afterwards.

#### 3.3.3. Methods and Results Comparison

**Figure 4:**
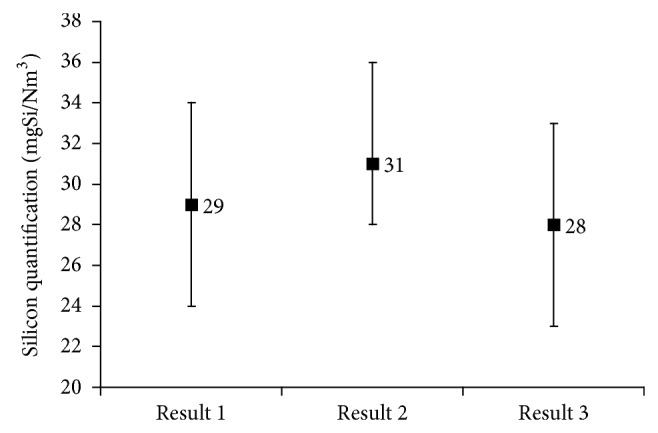
Comparison of experimental quantifications obtained by GC-MS (*Result 1*); ICP-OES (*Result 2*); and the theoretical ICP-OES by calculation (*Result 3*).

Figure 4 is a visual representation of the three results (experimental GC-MS; experimental ICP-OES; and theoretical ICP-OES) with their uncertainties.

Theoretically, the value of 1.5 ± 0.2 of the global correction factor is validated for the present analytical method as the theoretical ICP-OES value of 28 ± 5 mgSi/Nm^3^ (*Result 3*), simulated thanks to GC-MS data (%Si from L2 in total Si) and L2 individual response factor for ICP-OES analyses, is recovering in the range of values corresponding to experimental ICP-OES concentrations after correction (28 < mgSi/Nm^3^ LFG < 36,* Result 2*).

Experimentally, the conclusive similitude between the GC-MS result (29 ± 5 mgSi/Nm^3^ of LFG,* Result 1*) and the corrected experimental ICP-OES results (28 < mgSi/Nm^3^  LFG < 36,* Result 2*) confirms the necessity of an adjustment method, using a global correction factor of 1.5 ± 0.2.

By comparison of ICP-OES experimental (*Result 2*) and theoretical results (*Result 3*), it has also been proved that the analytical calculation is able to free ourselves from the analytical deviation linked to L2 presence in LFGs.

All three results added by their uncertainties are recovering each other. These results imply that ICP-OES method (using an adapted correction factor) provides the same results to GC-MS analysis in terms of total Si, which will allow performing more cost-effective analyses of total Si in biogases.

## 4. Conclusion

A liquid absorption in ethanol, considered more harmless and environmental friendly than methanol or other solvents, such as acetone, toluene, hexane, and heptane, minimizes the risks of biogas and LFG sampling. All VOSiCs are easily solubilized and then analyzed by ICP-OES which allows a global quantification of Si.

The only VOSiC identified as responsible for an analytical bias (L2), which could falsify consequently Si quantification, is absent from WWTPs DGs. Therefore, even if it has been verified, no global deviation needs to be measured for this type of gas, whatever the type of analysis. Finally, only the analysis of VOSiCs from LFGs (with a classical L2 percentage less than 25% of the total Si LFGs content), absorbed in ethanol, requires the use of a 1.5 ± 0.2 corrective factor to apply to the total Si level issued from the ICP-OES analysis.

The easy use of sampling and analysis protocols has revealed that the method uncertainty is acceptable for biogas treatment applications. A first step for Si total quantification in biogases is established and allows an access to a global biogas quality indicator. It can, in addition to a detailed occasional VOSiCs speciation, lead site managers in their equipment choices (type, size, etc.) thanks to a routine assessment. Results are given with a 15% uncertainty, equivalent to private laboratories ones.

Nevertheless, it remains possible to quantify separately polar VOSiCs from the less polar, by using water upstream from the ethanol bottles in charge of trapping the TMSol (thanks to the fact that 95% of Si biogases content is trapped in the first bottle and the 5 remaining percentiles in the second). In this case the methodology will still be applicable, as TMSol will be quantified separately in water over a TMSol calibration, so no bias occurs (*data not shown*) which is similar to its response factor of 1.1 in ethanol in comparison to L5 calibration.

## Figures and Tables

**Table 1:** Typical LFGs and DGs compositions in % of Si for each VOSiC by GC-MS and the total Si in mgSi/Nm^3^ biogas.

% Si	LFG A	LFG B1	LFG B2	LFG B3	LFG C	DG A	DG B
TMSol	26	11	24	6	36	1	1
L2	10	11	19	19	10	—	—
L3	1	7	1	1	1	—	2
L4	—	—	—	—	—	1	—
D3	2	19	3	3	3	—	2
D4	38	32	46	44	29	20	30
D5	23	19	7	6	21	78	65

Total Si content (mgSi/Nm^3^)	9	4	23	29	8	1	1

**Table 2:** Determination of the mean response factors observed by ICP-OES at −10°C for the 3 standards solutions at 2, 4, and 5 mgSi/L (L5 calibration).

% of Si/sample coming from L2	Mean response factor	RSD %
0	**1.0**	**2**
10	**1.3**	0
15	**1.4**	7
20	**1.6**	**4**
25	**1.7**	**0**

Median value (excluding 0% Si from L2)	**1.5**	—

**Table 3:** GC-MS analysis (LFG B3).

	mgSi/Nm^3^ biogas
TMSol	2
L2	6
L3	0
L4	<LD
L5	<LD
D3	<LD
D4	13
D5	2
TMS	6

Total	29

**Table 4:** ICP-OES analysis (LFG B3).

Bottles numbers	Si content
Bottle number 1 (mgSi/L EtOH)	6,0
Bottle number 2 (mgSi/L EtOH)	0,3
Bottle number 3 (mgSi/L EtOH)	<LD
Bottle number 4 (mgSi/L EtOH)	<LD
Si total (mgSi/L EtOH)	6,3
Si total (mg/Nm^3^ biogas)	**47**
Si total (mg/Nm^3^ biogas) adjusted by a global overestimation factor of:	
*1,3 *	36
***1,5***	**31**
*1,7 *	28
